# Registries for orphan drugs: generating evidence or marketing tools?

**DOI:** 10.1186/s13023-020-01519-0

**Published:** 2020-09-03

**Authors:** Carla E. M. Hollak, Sandra Sirrs, Sibren van den Berg, Vincent van der Wel, Mirjam Langeveld, Hanka Dekker, Robin Lachmann, Saco J. de Visser

**Affiliations:** 1 Department of Endocrinology and Metabolism, Amsterdam University Medical Centers, location Academic Medical Center, University of Amsterdam, F5-170, P.O. Box 22660, 1100 DD Amsterdam, The Netherlands; 2 Platform Medicine for Society at Amsterdam University Medical Centers, location Academic Medical Center, University of Amsterdam, Amsterdam, The Netherlands; 3 Division of Endocrinology and Metabolism, University of British Columbia, Vancouver, British Columbia Canada; 4 VKS, The Dutch patient association for Inherited Metabolic Diseases, Zwolle, Netherlands; 5 Charles Dent Metabolic Unit, National Hospital for Neurology and Neurosurgery, London, UK

**Keywords:** Registries, Orphan drugs, Real world data, Disease registries

## Abstract

Independent disease registries for pre-and post-approval of novel treatments for rare diseases are increasingly important for healthcare professionals, patients, regulators and the pharmaceutical industry. Current registries for rare diseases to evaluate orphan drugs are mainly set up and owned by the pharmaceutical industry which leads to unacceptable conflicts of interest. To ensure independence from commercial interests, disease registries should be set up and maintained by healthcare professionals and patients. Public funding should be directed towards an early establishment of international registries for orphan diseases, ideally well before novel treatments are introduced. Regulatory bodies should insist on the use of data from independent disease registries rather than company driven, drug-oriented registries.

## Orphans

Rare diseases (defined in the EU as affecting fewer than 1 in 2000 people) are individually rare but collectively common, affecting 6–8% of the population [1]. Orphan drugs, drugs for rare diseases, are developed under specific regulations in the EU, the United States and Canada with incentives to stimulate pharmaceutical companies to develop medicines for rare diseases. The idea is that investments in orphans would not be commercially attractive. However, several studies suggest that orphan drugs are associated with a higher return on investment than drugs licensed for common diseases resulting in intense pharmaceutical industry interest in rare diseases [2, 3]. While the number of treatable rare conditions is still relatively low, the orphan drug market is expanding at an annual growth rate of 11.2% and orphan drugs are expected to make up more than 18% of worldwide prescription drug costs by 2024 [4]. The majority are for oncology indications followed by cell- and gene-based therapeutics, the latter typically indicated for ultra-rare inherited diseases [5].

## The development process and registries

Orphan designation is granted to an applicant at the beginning of the drug development process based on the “medical plausibility” of the proposed active substance’s effect on a rare disease. However, conducting clinical studies to prove safety and effectiveness can be challenging due to small patient numbers and phenotypic variability. Because they are developed for rare diseases, orphan drugs may receive marketing authorization with limited safety and efficacy data [6]. Societal pressure for early access to these novel therapies is high. Orphan drug registration and authorisation is organized by authorities such as the Food and Drug Administration (FDA) in the US, Health Canada in Canada and the European Medicines Agency (EMA) in the EU. Data from disease registries can play a role in this process, helping to describe the natural history of the disease and identifying suitable clinical and surrogate endpoints for clinical trials [7].

## Post-registration and registries

Registries also have a role after marketing authorisation when regulatory authorities request data on the real-world effectiveness and/or safety profile of new drugs. Currently, the notion that real world data can be used in regulatory decision making is increasing [8]. Since clinical trials in rare diseases may be underpowered and of short duration, long-term data are needed to determine the optimal place of these treatments in disease management. Post-registration studies can be used to support recommendations in treatment guidelines. For instance, in the EU, the EMA may grant a conditional marketing authorisation, when the data provided in a marketing application is less comprehensive than would be accepted for a non-orphan indication [9]. The applicant is then obliged to provide comprehensive data to confirm the positive risk-benefit ratio of their product in commercial use but, when it is unlikely such data will be available, the EMA may still grant a marketing authorisation under exceptional circumstances. In these situations, a post-marketing registry may be required by the regulatory authorities or proposed by the applicant as part of the risk management plan.

## Real world data and industry

The development of registries to collect real world and life data for orphan drugs is largely driven by the requirements imposed by regulators on applicants for marketing authorisation. These requirements do not always result in the development of registries with open access to real-world data. Firstly, many of these registries (which are drug focused rather than disease-oriented) have been set up to fulfil marketing obligations. Engagement of patient representatives and healthcare professionals is very important to help identify the most clinically relevant outcome parameters and indicators of quality of life [7]. However, this input comes generally too late and is therefore insufficient to capture adequate information for biomarker research, natural history and optimal disease management, which will be necessary for evaluation of effectiveness and appropriate use. Secondly, regulatory agencies can request that data are collected following Good Clinical Practice standards, however, they are frequently unable to review whether these data are sufficiently consistent and relevant. Data sets may be incomplete making it impossible to distinguish subgroups of patients with different phenotypes and differential risk of suffering defined disease complications [10]. Thirdly, the operation of these industry-sponsored registries may result in conflicts of interests between pharmaceutical companies, healthcare professionals and patients. For instance, data from these registries can only be accessed and published with the support of the sponsor. These publications, based on data owned by the industrial sponsor, are then often used as the basis of clinical guidelines. Meetings of clinical experts are convened by the sponsor to draft guidelines (often facilitated by industry-hired medical writers) which depend heavily on expert opinion and systematic literature reviews of publications which are mostly based on registry data [11]. Because the data from industry funded registries may not be freely exchangeable or accessible for analysis by third parties, there is a risk that publications from registries, and the guidelines that arise from them, may be biased [12].

## Orphan drugs for non-oncology indications

Orphan drugs for rare (non-oncology) indications account for 88 of the 129 currently authorized orphan drugs [13] and, as is typical in rare diseases, no other specific treatments were available at time of initial marketing authorisation. In almost 30% of orphan drug approvals (Table 1), the orphan drug was authorised by EMA either with conditional approval or approval under exceptional circumstances, usually resulting in the set-up of a registry. In addition, in half of the ‘regularly’ approved cases a registry was launched. In total, 62,5% of approvals coincided with registry establishment.

**Table 1:** Approval types and registries used for post-marketing regulatory purposes for orphan (non-oncology) drugs from 2000 to 2019. (data from the EU PAS register [14], European public assessment reports (EPARs) [13, 15] or publicly available information)

Approval type	Subtotal	Registry not part of approval	Registry part of approval
Conditional approval	9	1 (11,1%)	8 (88,9%)
Exceptional circumstances	16	0 (0%)	16 (100%)
No conditional approval or exceptional circumstances	63	32 (50,8%)	31 (49,2%)
**Total**	**88**	**33 (37,5%)**	**55 (62,5%)**

## Registries and industry

**Fig. 1:**
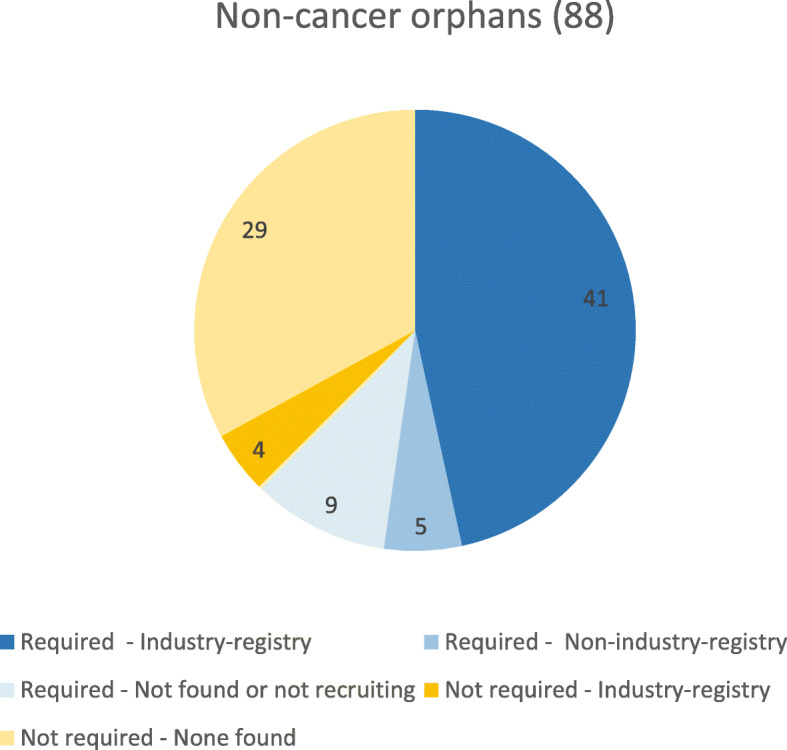
Origin of registries (data from the Encepp PAS database, risk management plan, European public assessment report (EPAR) or publicly available information) [13–15]

The majority of the registries requested or suggested in the approval process of orphan drugs (41 of 55) have been initiated and funded by industry (Fig. 1). In only 5 cases non-industry registries were set up for regulatory approval (cystic fibrosis for two products, spinal muscular atrophy, Cushing Syndrome, and Hemophilia B). For 4 drugs, an industry-initiated registry not mandated for regulatory purposes was established. Almost all these industry registries have a set reporting date and it is not clear what will happen to the registry after that date.

## Lysosomal storage disorders

Over the last 25 years a number of new drugs have been licensed for use in lysosomal storage disorders (LSD’s). Many of these have associated post-marketing registries to address long-term safety and effectiveness outcomes. These registries provide an example of how these regulatory requirements are set up and used in practice (Table 2).

**Table 2:** Overview of the LSD disease registries sponsored by pharmaceutical industry

Disease	Registry	Year established	Sponsor	Patients enrolled (N)
LAL-D	Lysosomal Acid Lipase (LAL) Deficiency Registry (ALX-LALD-501)	2012	Alexion	1000
Fabry	Fabry Disease Registry	2001	Genzyme, a Sanofi Company	9000
	Fabry Outcome Survey (FOS)	2001	Shire	4000
Gaucher	International Collaborative Gaucher Group (ICGG) Gaucher Registry	1991	Genzyme, a Sanofi Company	12,000
	Gaucher Disease Outcome Survey (GOS)	2010	Shire	1257
MPS I	Mucopolysaccharidosis I (MPS I) Registry	2003	Genzyme, a Sanofi Company	1500
MPS II	Hunter Outcome Survey (HOS)	2005	Shire	2000
MPS IVType A	A Multicenter, Multinational, Observational Morquio A Registry Study (MARS)	2014	BioMarin Pharmaceutical	583
MPS VI	Mucopolysaccharidosis (MPS) VI Clinical Surveillance Program (CSP)	2005	BioMarin Pharmaceutical	200
MPS VII	Mucopolysaccharidosis VII Disease Monitoring Program	2018	Ultragenyx Pharmaceutical Inc	35
Pompe	Pompe Disease Registry	2004	Genzyme, a Sanofi Company	2000
	Alglucosidase Alfa Pompe Safety Sub-Registry	2015	Genzyme, a Sanofi Company	110
	Pompe Lactation Sub-Registry	2012	Genzyme, a Sanofi Company	5
	Pompe Pregnancy Sub-Registry	2011	Genzyme, a Sanofi Company	20
CLN2	Examining Developmental Outcomes of Children Diagnosed With CLN2 Disease	2018	Jessica Scherr / Biomarin Pharmaceutical	30

LSD’s are a group of ultra-rare disorders with a prevalence ranging from less than 1 in 100.000 up to 1 in 30.000 [16]. These disorders are inborn errors of metabolism caused by a deficient enzyme, with in general a wide array of phenotypes. The prototype treatment is enzyme replacement therapy (ERT): intravenous administration of a recombinant enzyme, which supplements the deficiency to degrade stored macromolecules.

Gaucher disease was the first LSD shown to be treatable by enzyme replacement. Treatment with Ceredase, a placental derived enzyme, resulted in reversal of massive hepatosplenomegaly and cytopenias and prevented severe bone complications: a landmark achievement. Genzyme corporation (now a Sanofi company) entered this ultra-orphan area under the USA orphan drug act, granting them 7 years of market exclusivity. At that time Ceredase was the most expensive medicine ever: treatment of a 50 kg patient with the licensed dose came in at a cost between 50.000 and 500.000 USD per year [17]. However, it soon became clear that, as a result of variability in phenotypes, not all patients needed treatment and those that did could often be treated with lower doses than the licensed dose [17].

## Enter registries

Following debates on dosing and differences in the natural disease course, a 1995 Health Technology Assessment meeting resulted in a recommendation to seek better evidence to support the appropriate use of this costly therapy [18]. Also for this purpose, Genzyme set up a global registry in 1991 containing data on both treated and untreated patients: run and financed by Genzyme, analyses performed by Genzyme and publications supported by Genzyme. An international board of Gaucher treating physicians helped to operate the registry: meetings were held, with key opinion leaders, paid by Genzyme, discussing treatment goals and diagnostic algorithms. These discussions resulted in manuscripts, (written by a medical writer hired by Genzyme), that were published in peer reviewed journals. Interestingly, none of the Gaucher Registry publications reported on limitations of treatment with enzyme replacement therapy [19]. A similar approach for Genzyme’s (now Sanofi) oral drug for Gaucher (Eliglustat) resulted in a treatment algorithm publication, without mentioning the possibility of the patient having mild disease status that does not require treatment [20].

## New drug, new registry

When their competitor Shire (now Takeda) launched a similar ERT in the EU in 2014, another global post-marketing registry for Gaucher disease was started (initiated in 2010) to address the same issues of unmet medical needs, diagnostic challenges and treatment goals. Both companies financially supported separate sponsored symposia, round tables for educational purposes and investigator-initiated studies. These frequently led to recommendations for early initiation of therapy or population screening, including newborn screening (NBS) to find new patients. A pilot NBS study on NBS for a panel of lysosomal storage disorders did not identify any early onset phenotypes which might have benefited from pre-symptomatic initiation of therapy [21].

## Fabry disease

Following the success of ERT in Gaucher disease, the second disorder that was targeted was Fabry disease, a disease that can cause neurologic, renal and cardiac dysfunction. In this case, Genzyme and Shire were both able to market their enzymes at the same time in the EU at a mean similar price of EUR 200.000 per patient per year. Two separate global registries (initiated in 2001) were mandated by the EMA to address open questions concerning effectiveness and safety. There has been no exchange of data or collaborative analysis of data from the two registries. Separate, sponsored groups have addressed the same questions following the pattern described for Gaucher disease. Unfortunately, ERT for Fabry disease has proven to be less effective in preventing complications than ERT for Gaucher disease. The diversity of phenotypes, lack of knowledge about differences in the natural disease course in the different patient groups and the development of treatment interfering neutralizing antibodies in many patients has hampered our ability to draw any robust conclusions [22]. There remains real uncertainty about whom to treat, at what dose and for how long. It is clear that the registries were not set up to be able to answer these questions, since crucial data e.g. on patients’ disease phenotype, were usually missing and results from the different antibody assays used by the companies could not be compared [23].

## Independent registry for Fabry disease

These uncertainties have led to some independent initiatives, including the Canadian Fabry Disease Initiative and a European database, that eventually led to the generation of some independent guidelines and recommendations for diagnosis and treatment start and stop criteria [23, 24]. However, the long-term uncertainty about the effectiveness of treatment has impacted reimbursement decisions: in the Netherlands, in 2011, a provisional decision to stop reimbursement of both ERT’s for Fabry, and ERT for Pompe disease, based upon the poor quality of data, led to national uproar. Not only was the quality of the data available insufficient to make informed decisions, but the existence of the EMA-mandated post-marketing registries hampered the set-up of independent databases: healthcare professionals were not keen to contribute to yet another database for which there was no financial support for data monitoring, meetings and publications. In fact, by putting the pharmaceutical industry in charge, regulators have accepted that companies become involved in the set-up of guidelines, which carries a risk for biased recommendations [11, 12].

## Lysosomal acid lipase deficiency

Following the Gaucher HTA meeting and call for natural history studies early in the process of drug development, companies have become increasingly interested in pre-marketing data acquisition. Examples of this are the initiatives for pre-marketing data collections for lysosomal acid lipase deficiency (LALD), an ultra-rare LSD with variable presentation which in its severe forms may lead to liver failure. The manufacturer of ERT for this disorder, Synageva, later acquired by Alexion, sponsored publications on untreated patients in the pre-marketing phase, pushing the message through their key opinion leaders that LALD is a devastating disorder, with a progressive course, in all patients [25]. Thus, once diagnosed, patients would need to be treated with their product, sebelipase alfa, that comes at a price of up to 800.000 Euro per patient per year. However, no long-term follow-up of untreated, mildly affected patients has ever been presented. Anecdotal experience suggests that patients at the milder end of the spectrum may not benefit from therapy at all. In addition, it is also not clear that long-term complications can be prevented by treating patients with severe or advanced disease. However, physicians seeing a single patient or family will rely on published recommendations, and end up treating their patients. It is no surprise that Alexion has set up a Global LAL Deficiency Registry in 2012 to document the use of their product.

## From drug- to disease registry

The current variable approach to real-world data collection with a predominance of industry owned, drug-oriented patient registries is not fit for purpose and should be reassessed. Since several stakeholders contribute to the current system, recommendations for change should be addressed to all. Firstly, there needs to be change in the mindset of healthcare professionals towards the ownership of patient data. This implies that they should be pro-active in the set-up of independent, sustainable disease registries, which are then governed by patients and healthcare professionals together [26]. These databases should contain all data needed to gain a full picture of the natural history and optimal management of disease, as well as the data needed to answer the very specific questions about safety and outcomes asked by regulators and industry. Secondly, pharmaceutical companies should accept the call for generation of post-marketing evidence for healthcare that is free of commercial influences. Recently, an international group of scientists has published pathways to independence, clearly pointing out that this is needed for trustworthy evidence [27].

Thirdly, regulatory authorities have launched several initiatives to use post-marketing evidence generation for regulatory decision-making [28]. This should not be undertaken without healthcare professionals and patients as necessary partners and should use data from independent patient registries to avoid conflicts of interest [29].

## Registry funding

Lastly, governments and/or regulatory authorities should make commitments to fund accessible patient/disease registries, which will then be independent of industry [27]. For rare diseases, registries should be initiated long before a novel treatment receives marketing authorisation. If there isn’t a disease registry in place at the time of orphan designation for a new drug, then that would be a good time to start one. Once registries have been established, then ongoing structural funding can come from other sources, including industry, in the form of mandatory fees which could be linked to clinical trial registration and market authorisation. Such fees should not raise the costs of orphan drugs, since industry sponsored registries would no longer be needed. In addition, public funding could be made available for investigator-initiated studies e.g. on appropriate use, conducted by centres of expertise, who will maintain the registry. EMA/FDA and/or other regulatory bodies including HTA bodies making reimbursement decisions can further empower independent registries by directing post (or even pre) marketing research to these registries.

## Conclusion

The undue influence on clinical practice of the use of post marketing registries as marketing tools is underestimated. The virtual absence of independent registries for rare diseases results in unwanted conflicts of interest. Health care professionals and patient organizations, operating independently of industry, should take responsibility for providing and generating independent data which can be used to (re) evaluate registration/reimbursement decisions and guide optimal patient management in the challenging field of treating patients with rare diseases.

## Data Availability

The datasets used and/or analysed during the current study are available from the corresponding author on reasonable request.
